# Effectiveness of the Mobile-Based Diabetes Little Helper Video Intervention on Medication Adherence Among Older Adults Living With Type 2 Diabetes Mellitus, Henan, China: Randomized Controlled Trial

**DOI:** 10.2196/78731

**Published:** 2026-02-17

**Authors:** Meng Wang, Khuan Lee, Hui Zhu Thew, Siti Noorkhairina Sowtali, Qiuhuan Jiang, Zhengyan Zhao, Yu Liu, Poh Ying Lim

**Affiliations:** 1 Department of Nursing, Faculty of Medicine & Health Sciences Universiti Putra Malaysia Selangor Malaysia; 2 Department of Family Medicine, Faculty of Medicine & Health Sciences Universiti Putra Malaysia Selangor Malaysia; 3 Department of Professional Nursing Studies, Kulliyyah of Nursing International Islamic University Malaysia Pahang Malaysia; 4 Henan Provincial People's Hospital Zhengzhou China; 5 Zhengzhou Seventh People's Hospital Zhengzhou China; 6 The Second Mobile Contingent Hospital of the Chinese People's Armed Police Forces Wuxi China; 7 Department of Community Health, Faculty of Medicine & Health Sciences Universiti Putra Malaysia Selangor, Serdang Malaysia

**Keywords:** older adults, video, type 2 diabetes mellitus, medication adherence, randomized controlled trial

## Abstract

**Background:**

Medication adherence is vital for older adults living with type 2 diabetes mellitus (T2DM), but it remains low and needs improvement. Current interventions have limited effectiveness, while video-based interventions show promising potential for enhancing adherence.

**Objective:**

To evaluate the impact of the “Diabetes Little Helper” video intervention, developed based on the information–motivation–behavioral skills model, on improving medication adherence in older adults living with T2DM in Henan.

**Methods:**

This parallel-group randomized controlled trial was conducted in 2 hospitals in Zhengzhou, involving 68 patients from each hospital. The intervention group (IG) received standard care plus the video intervention for one month, while the control group (CG) received only standard care. The primary outcome was medication adherence, and secondary outcomes included medication knowledge, attitude, behavior, belief, and social support. Data were collected at baseline, postintervention, and at 3-month follow-up. Intention-to-treat analysis and the last observation carried forward method were applied for missing data, with the generalized estimating equation model used for effect assessment (*P*<.05 considered statistically significant).

**Results:**

The average age of participants in the IG was 67.5 (SD 8.0) years, while in the CG, the average age was 66.0 (SD 7.0) years. Sex distribution was nearly identical, with 51.5% (n=35) of participants in the IG and 50.0% (n=34) in the CG being male. After the intervention, the IG and CG had retention rates of 95.6% (n=65) and 97.1% (n=66), respectively. At the 3-month follow-up, the retention rates for the IG and CG were 92.6% (n=63) and 92.2% (n=62), respectively. Both postintervention (β=4.956, 95% CI 3.702-6.210, *P*<.001) and at the 3-month follow-up (β=3.691, 95% CI 2.379-5.003, *P*<.001), medication adherence score for the IG was significantly higher than that for the CG. In addition, the IG showed significant improvements in secondary outcome, with medication knowledge (β=11.592, 95% CI 6.923-16.260, *P*<.001), attitude (β=5.467, 95% CI 4.531-6.763, *P*<.001), behavior (β=4.176, 95% CI 3.220-5.133, *P*<.001), and belief (β=2.882, 95% CI 1.990-3.775, *P*<.001) compared with the CG postintervention. However, there was no statistically significant difference in the secondary outcome of social support (β=0.008, 95% CI –1.834 to 2.011, *P*=.928).

**Conclusions:**

The Diabetes Little Helper video intervention effectively improved medication adherence in older adults living with T2DM in Henan, highlighting the potential of digital health tools for managing chronic diseases in older adult populations.

**Trial Registration:**

Chinese Clinical Trial Registry ChiCTR2400083282; https://www.chictr.org.cn/showprojEN.html?proj=214847

## Introduction

With the continuous improvement of economic levels, changes in lifestyle, and the accelerated process of population aging, the prevalence of diabetes mellitus (DM) has been steadily increasing, becoming a significant global public health challenge [1,2]. In 2021, the number of people aged 20-79 years with DM worldwide was approximately 536.6 million, and it is projected to rise to 783.2 million by 2045 [3]. The older adults, due to factors such as physiological decline, metabolic imbalance, and the coexistence of chronic diseases, represent a high-risk group for DM. In 2019, the number of people aged 65 years and older with DM worldwide reached 135.6 million, and it is expected to increase to 276.2 million by 2045 [4].

As one of the countries with the highest burden of DM, the trend of DM prevalence in China is consistent with the global pattern. The number of people aged 20-79 years with DM in China increased from 88.7 million in 2020 to over 108.4 million [5]. Among these, type 2 diabetes mellitus (T2DM) is the predominant form, accounting for 90%-95% of all DM cases, with the older adults being the primary affected group [6-9]. T2DM significantly shortens the lifespan of patients and can lead to severe complications, including cardiovascular diseases, kidney disease, retinopathy, and neuropathy, potentially resulting in organ failure [10-15]. Due to the long duration of the disease and the presence of multiple complications, older adults living with T2DM are at higher risk of developing complications such as diabetic foot, stroke, and nephropathy [16,17]. Medication treatment is crucial in the management of T2DM, and its effectiveness is highly dependent on patient adherence to medication, that is, whether patients follow the prescribed treatment regimen consistently [18,19]. Studies have shown that patients with high medication adherence not only experience more stable disease control but also significantly reduce mortality, hospitalization rates, and health care costs [20-22]. However, in China, more than half of older adults living with T2DM experience poor medication adherence [23,24], which severely limits the long-term effectiveness of DM management and urgently calls for effective intervention strategies. Research indicates that various factors contribute to poor medication adherence, including age [25], economic burden [26], and multiple medications [27]. To improve medication adherence among older adults, various intervention strategies have been attempted both domestically and internationally, including health management based on multidisciplinary collaborative models [28], face-to-face health education [29], mobile app support [30], and motivational interviewing [31]. However, these approaches generally face challenges such as high implementation costs, complex operations, and a strong reliance on health care resources. Furthermore, most lack systematic theoretical models, making them difficult to adapt to older adults with lower educational levels [32,33]. The information–motivation–behavioral skills (IMB) model provides a theoretical foundation for explaining and predicting individual health behaviors and has been widely applied in chronic disease self-management research [34,35]. Existing studies have confirmed that the IMB model can effectively predict medication adherence among older adults living with DM [36,37], offering scientific guidance for the development of intervention strategies. In recent years, video intervention has emerged as a new method of health communication. Existing studies have shown that videos not only effectively capture attention but also facilitate the understanding and retention of information [38]. Particularly within older adult populations, multimedia interventions have been demonstrated to overcome the limitations of traditional educational methods, improving the acquisition of health knowledge and enhancing patients’ self-management capabilities [39,40]. Videos, delivered through convenient devices such as mobile phones, enable patients to view them anytime and anywhere, further reinforcing learning outcomes and improving medication adherence [41]. However, there is currently no research using video interventions to improve medication adherence among older adults living with T2DM. Therefore, exploring the application of IMB-based video interventions in this population and their potential to enhance medication adherence holds significant theoretical and practical importance. Based on this, we hypothesize that the Diabetes Little Helper video intervention will significantly improve medication adherence compared with standard care.

## Methods

### Ethical Considerations

This study received ethical approval from the Institutional Review Board of Universiti Putra Malaysia (JKEUPM-2023-1279) and Henan Provincial People’s Hospital (20240107), and was registered with the Chinese Clinical Trial Registry (ChiCTR2400083282). Before data collection, the researcher obtained approval from the hospital and coordinated with relevant departments to ensure informed consent from participants and guide them to scan the QR code to follow the “Diabetes Little Helper” WeChat public account. All participant information was kept strictly confidential, and the questionnaire data were anonymized after collection to ensure that participants’ identities could not be directly or indirectly identified. This study does not involve any monetary compensation or incentives for participants.

### Study Design and Setting

This study was a parallel-group, cluster-randomized controlled trial (RCT) recruited older adults from 2 hospitals in Zhengzhou, Henan Province, China (Henan Provincial People’s Hospital and Zhengzhou Central Hospital). Both hospitals are large, tertiary-level public general hospitals with an annual outpatient volume exceeding 1.1 million visits. Each hospital has over 70 clinical departments, including endocrinology, respiratory medicine, and cardiology, offering comprehensive medical services and a sufficient patient base for recruitment. The study spanned from April 2024 to June 2025, encompassing participant recruitment (from April to June 2024), intervention delivery, data collection, analysis, and manuscript preparation. The study was conducted and reported in accordance with the CONSORT (Consolidated Standards of Reporting Trials; checklist provided in Multimedia Appendix 1) guidelines. Further details of the study design and methodology are available in the published protocol in *BMJ Open* [42]. This study followed all specified procedures without any deviations from the protocol.

### Participants

This study involved older adults diagnosed with T2DM who attended endocrinology outpatient clinics at 2 selected hospitals. Eligible individuals were identified from the outpatient population of each hospital and enrolled through simple random sampling. Participants must meet the following inclusion criteria: aged 60 years or older, diagnosed with T2DM [43], Chinese nationality, and able to understand Chinese, currently receiving at least one oral antidiabetic medication with a hemoglobin HbA_1c_ level of ≥7.0%, no cognitive impairment, and either the participant or their family member owns a smartphone and is able to use WeChat. Exclusion criteria include having severe comorbidities affecting major organs such as the heart, lungs, or kidneys, impairment in communication abilities, or participating in another study.

This cluster RCT study involves 2 hospitals, which were randomly assigned in a 1:1 allocation ratio to the control group (CG) and intervention group (IG) using a random number generator. Both hospitals used simple random sampling and a parallel design, recruiting 68 older adults living with T2DM. Randomization was conducted by the research coordinator using a computer-generated random number sequence, with concealed allocation to prevent selection bias. The CG received standard nursing care, while the IG received additional video intervention. Due to the visible nature of the video intervention, the study was conducted as an open-label trial, meaning blinding was not feasible for the participants or executors. However, evaluators and statistical analysts remained blind to minimize bias. To minimize contamination, participants were selected from separate hospitals, and communication between the groups was restricted. After a 3-month follow-up, the CG received the same video intervention as the IG to ensure equal treatment.

The sample size for this study was calculated using the 2-mean sample size formula [44], based on the mean and SD of medication adherence for both CG and IG at postintervention and 3-month follow-up (29.15, SD 3.10; 31.27, SD 3.35; and 26.25, SD 2.01; 30.19, SD 2.41) [45,46]. Assuming a 2-sided test with a type I error rate (α) of 0.05, a power (1-β) of 80%, an 80% retention rate, and a design effect of 1.5, the required total sample size was calculated to be 68 participants per group.

### Diabetes Little Helper Intervention Development

A health education intervention program based on the IMB theory model was developed for this study. The research team conducted 3 rounds of expert consensus meetings involving a multidisciplinary expert panel, which included 2 nursing experts with extensive clinical experience and expertise in video production, a biostatistician with a background in statistics for health interventions, and a family medicine specialist with expertise in DM management. The team collaboratively established a comprehensive educational framework covering 8 core topics related to DM medication management. These topics include proper medication storage and transportation, methods for identifying medication quality, disease management strategies, the importance of medication adherence, key medication precautions, common misconceptions about medications, self-management empowerment for older adults living with DM, and guidelines for blood glucose monitoring. For each topic, a 2-5 minute educational animation was developed using the “WanCai Animation Master” tool to visualize professional medical knowledge into easy-to-understand dynamic images [47]. Table 1 shows the Diabetes Little Helper video intervention topic and plan.

**Table 1:** Topics and intervention plan of the Diabetes Little Helper video program for older adults living with type 2 diabetes mellitus in Henan, China.

Number	Week	Day of the week	IMB^a^	Topic	Video time
1	One	Tuesday	I	Storage & Traveling with Hypoglycemic Medications	3 min 31 s
2	One	Friday	I	How to Check Your Medications	3 min 20 s
3	Two	Tuesday	I	What to Do During Sick Days	2 min 57 s
4	Two	Friday	M	Importance of On-Time Medication	2 min 15 s
5	Three	Tuesday	M	Precautions for Taking Hypoglycemic Medication	1 min 53 s
6	Three	Friday	M	Correct Understanding of Hypoglycemic Medications	2 min
7	Four	Tuesday	B	Self-Empowerment & Support for Older Adults Living with DM	4 min 30 s
8	Four	Friday	B	Regular Blood Glucose Monitoring	3 min 6 s

^a^IMB: information–motivation–behavioral skills.

Five nursing experts were invited to assess the content validity of the program based on the standards developed by Siti Noorkhairina et al [48]. These standards were specifically designed for evaluating video-based educational materials and cover various aspects of content quality, including media characteristics (title relevance and audio clarity), educational design (plot logic and age-appropriate language), and technical specifications (appropriate duration and information density). The Siti Noorkhairina et al standards provide a comprehensive framework for assessing the effectiveness of educational videos, ensuring that the content is clear, relevant, and engaging for the target audience. Experts rated 24 evaluation items based on these criteria to determine the content validity of the program. A 4-point Likert scale was used for the ratings. The Item-level Content Validity Index (I-CVI) for each of the 24 items was calculated by determining the proportion of experts who rated the item as relevant, with scores of 3 or 4 indicating relevance. For calculation, scores of 3 and 4 were recoded as “1.00” (indicating relevant), and scores of 1 and 2 were recoded as “0” (indicating non-relevant). The I-CVI for each item was then calculated as the proportion of experts who rated the item as “relevant”. A value of 1.00 for the I-CVI indicates unanimous agreement among the experts that each item was deemed relevant. The Scale-level Content Validity Index (S-CVI) was calculated by averaging the I-CVI scores across all items. A value of 1.00 for the S-CVI indicates that the overall scale was deemed fully valid by all experts. These results suggest that the content of the program was considered highly relevant and appropriate by all expert raters. Ten older adults living with T2DM were invited to participate in a face validity test, which focused on information reception indicators such as language comprehensibility, visual appeal, and pacing control. Based on patient feedback, the audiovisual elements of the videos were optimized.

### Diabetes Little Helper Intervention Implementation

The intervention was implemented using a “standard care + digital health education” model, delivering content through the WeChat public account “Diabetes Little Helper.” During the 4-week intervention period, educational videos were scheduled to be released at 8 AM every Tuesday and Friday, with a total of 8 episodes. Patients could watch the videos at their convenience.

To ensure that patients successfully watched the intervention videos and to improve intervention quality, the research team took the following measures: (1) real-time monitoring of video viewership to track whether patients watched the videos on time, ensuring timely completion and optimizing the intervention strategy, (2) weekly WeChat reminders for patients to watch the new videos, along with contact information for researchers to provide immediate assistance, ensuring patients received the necessary information and encouraging participation, (3) weekly phone follow-ups to check patients’ video-watching progress, particularly focusing on those who missed videos, researchers would inquire about reasons for nonviewing and provide personalized help to ensure patient involvement, (4) personalized support for patients with special circumstances or frequent video nonviewing, including simple viewing guides and remote technical support to resolve technical issues, ensuring patients could watch the videos without difficulty.

### Development of a Questionnaire to Evaluate Medication Adherence, Knowledge, Attitude, Behavior, Belief, and Social Support

A self-developed questionnaire was used to collect data from participants, covering 8 aspects. A detailed overview of all outcome measures used in this study can be found in the study protocol [42]. The measures analyzed in this manuscript are described below.

### Sociodemographic and Disease-Related Characteristics

This study collected sociodemographic characteristics (such as age, sex, and marital status) as well as disease-related variables (such as the duration of T2DM, family history, and adverse medication reaction).

### Medication Adherence

Medication adherence is assessed using the Self-Efficacy for Appropriate Medication Use Scale [49], which includes 13 items, rated on a 3-point Likert scale, with a total score range of 13 to 39. Higher scores indicate better medication adherence. The Cronbach α value of the scale is 0.934 [49,50].

### Medication Knowledge, Attitude, and Behavior

The medication knowledge, attitude, and behavior questionnaires are used to measure patients’ knowledge, attitudes, and behaviors regarding medication. The test-retest reliability for each dimension ranges from 0.854 to 0.920, with an overall test-retest reliability of 0.868 [51]. The knowledge questionnaire consists of 17 items, with a total score range of 0 to 17, where higher scores indicate a greater level of medication knowledge. The medication attitude questionnaire includes 10 items, with a total score range of 10 to 50, where higher scores reflect a more positive attitude toward safe medication use. The medication behavior questionnaire consists of 13 items, with scores ranging from 9 to 40, where higher scores indicate safer medication behaviors.

### Medication Belief

The medication belief questionnaire is used to assess the personal medication beliefs of older adults living with DM [52]. The questionnaire consists of 10 items, divided into 2 dimensions, medication necessity and medication concern, with scores ranging from 1 to 5. Higher scores indicate stronger medication beliefs. The Cronbach α value for the questionnaire is 0.724, with Cronbach α values of 0.749 and 0.796 for the 2 dimensions, respectively.

### Social Support

The social support questionnaire is used to assess the support patients receive across multiple dimensions [53]. This scale includes 4 dimensions, with a total of 19 items, and the total score ranges from 19 to 95. Higher scores indicate higher levels of social support among the older adults. The questionnaire demonstrates good reliability and validity, with a Cronbach α value of 0.803 and a content validity index of 0.958 [53].

To ensure the content validity of the questionnaire assessing medication adherence, knowledge, attitude, behavior, belief, and social support, 5 experts reviewed it to confirm that the content aligned with the research objectives and that the questions were relevant and representative of the study. Based on expert feedback, some questions were adjusted to ensure they accurately measured the intended content. The I-CVI and the S-CVI of the questionnaire were both calculated to be 1, indicating strong content validity. Additionally, to assess the face validity of the questionnaire, it was presented to 10 older adults living with T2DM, who provided feedback on the clarity and appropriateness of the language used in the questions. Based on their feedback, the wording of some questions was revised for better clarity and suitability. The revised questionnaire was found to be relevant, user-friendly, and suitable for this population.

### Data Collection

Data collection occurred at 3 time points: baseline, postintervention, and 3-month follow-up. The questionnaire was available in both paper and electronic formats. Baseline data were collected between April and June 2024. Researchers conducted on-site checks to ensure the quality of questionnaire completion, confirming that all questions were fully answered and met the required standards. For older adult participants who had difficulty filling out or understanding the questions, standardized instructions were provided to help them interpret the content and complete the questionnaire correctly. During the postintervention and follow-up periods, the electronic questionnaire was sent to participants via a WeChat official account, with researchers assisting through WeChat or phone calls to ensure a high response rate and data quality. After collecting the questionnaires, researchers reviewed and processed any inconsistent or missing data to ensure completeness. Data were entered by 2 research assistants and imported into SPSS (version 29.0; IBM Corp) software for analysis. To reduce entry errors, all data were proofread twice after input to ensure consistency with the original questionnaires. Data from the electronic questionnaires were directly imported into SPSS.

### Data Analysis

Data analysis was performed using SPSS Statistics, following the intention-to-treat principle. Missing data were handled using the last observation carried forward (LOCF) method. A significance level was set at *P*<.05. Histograms with normal curves were used to assess the normality of continuous variables. For normally distributed variables, data are expressed as mean (SD), while not normally distributed variables are presented as median and IQR. Categorical variables are reported as frequencies and percentages (%).

For baseline group comparisons, the Pearson chi-square test and chi-square test with continuity correction were applied for categorical variables. Independent samples *t* test and Mann-Whitney *U* test were used for continuous variables. To assess the intervention effects, generalized estimating equation models with an unstructured working correlation matrix were used to analyze changes in medication adherence, knowledge, attitude, behavior, belief, and social support scores. The models compared the changes between the IG and CG at baseline, postintervention, and at the 3-month follow-up, with time, group, and their interaction as independent variables. The interaction term was used to assess whether there were different changes between the groups at different time points. Significant differences between the groups were adjusted for as covariates in the final model.

## Results

### Participant Flow

**Figure 1:**
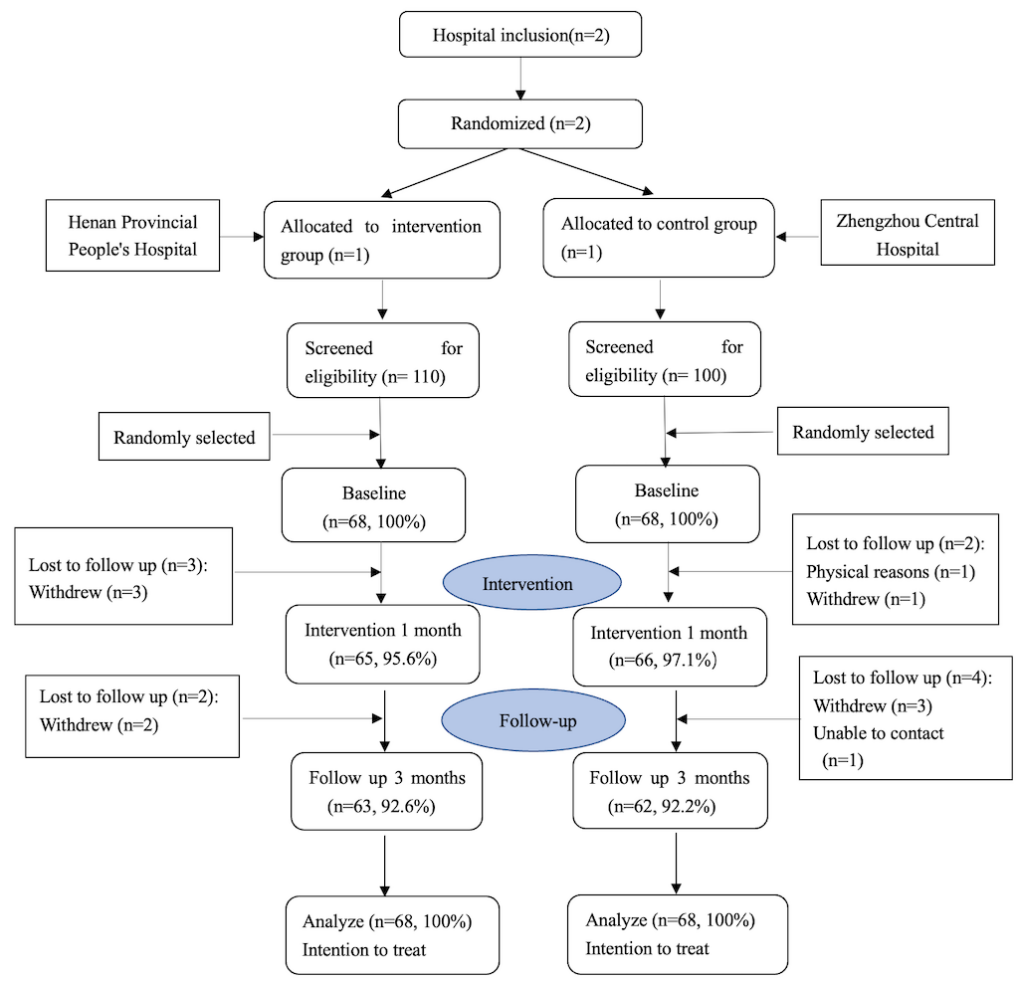
CONSORT (Consolidated Standards of Reporting Trials) flow diagram for a cluster randomized controlled trial on the effectiveness of the Diabetes Little Helper intervention video among older adults living with type 2 diabetes mellitus.

The IG consisted of 68 participants randomly selected from a pool of 110 eligible patients in one hospital, while the CG included 68 participants randomly selected from a pool of 100 eligible patients in the other hospital. Compared with the web-based questionnaire, 26 (29.0%) participants in the IG and 20 (38.0%) participants in the CG chose to complete the paper questionnaire. Both groups had an initial retention rate of 100% (n=68). After the intervention, 3 participants dropped out of the IG, leading to a retention rate of 95.6% (n=65). In the CG, one participant did not participate due to health issues, and another withdrew, resulting in a retention rate of 97.1% (n=66). Following a 3-month follow-up, 2 participants left the IG, giving a retention rate of 92.6% (n=63). In the CG, 3 participants withdrew, and one could not be contacted, leading to a retention rate of 92.2% (n=62). Figure 1 shows the data collection flow according to the CONSORT guidelines.

### Participant Characteristics

#### Sociodemographic and Disease-Related Characteristics Between the IG and CG at Baseline

At baseline, a comparison of sociodemographic and disease-related characteristics between the IG and CG showed no statistically significant differences (*P*>.05). The sex distribution was nearly identical, with 51.5% (n=35) in the IG and 50.0% (n=34) in the CG being male. Most participants lived in urban areas (IG: 38/68, 55.9%; CG: 42/68, 61.8%) and resided with family members (IG: 62/68, 91.2%; CG: 60/68, 88.2%). A large portion of participants were married (IG: 50/68, 73.5%; CG: 52/68, 76.5%). All participants had children, and education levels were primarily at the junior high school (IG: 22/68, 32.4%; CG: 10/68, 14.7%) and high school (IG: 21/68, 30.9%; CG: 22/68, 32.4%) levels. Additionally, a significant portion of participants did not use smartphones independently (IG: 15/68, 22.1%; CG: 10/68, 14.7%). The mean and SD for BMI and the duration of T2DM were as follows: IG 24.04 (SD 3.475), CG 24.08 (SD 3.428); IG 14.76 (SD 8.381), CG 14.19 (SD 7.658). Most participants used oral medications (IG: 37/68, 54.4%; CG: 35/68, 53.3%), and the proportion of family members without diabetes was similar (IG: 26/68, 33.3%; CG: 23/68, 29.5%). The most common adverse reactions were hypoglycemia (IG: 21/68, 30.9%; CG: 16/68, 26.5%) and digestive system issues (IG: 13/68, 10.1%; CG: 10/68, 14.7%). Details are provided in Table 2.

**Table 2:** Comparison of sociodemographic characteristics between the intervention group (IG) and control group (CG) at baseline among older adults living with type 2 diabetes mellitus (T2DM) in Henan, China (N=136).

Variable				IG (n=68)		CG (n=68)		Chi-square (*df*)^a,b^		*t* test (*df*)^c^		Z^d^		*P* value
Age (years), median (IQR)				67.5 (63.3-71)		66.0 (69.8-63)		—^e^		—		–0.901^d^		.37
**Sex, n (%)**								0.029 (1)^a^		—		—		.86
	Male			35 (51.5)		34 (50.0)								
	Female			33 (48.5)		34 (50.0)								
**Residence, n (%)**								0.486 (1)^a^		—		—		.49
	Countryside			30 (44.1)		26 (38.2)								
	City			38 (55.9)		42 (61.8)								
**Marital status, n (%)**								2.039 (2)^a^		—		—		.36
	Married			50 (73.5)		52 (76.5)								
	Divorced			3 (4.4)		6 (8.8)								
	Widowed			15 (22.1)		10 (14.7)								
**Living arrangement, n (%)**								0.319 (1)^a^		—		—		.57
	Living alone			6 (8.8)		8 (11.8)								
	Living with family			62 (91.2)		60 (88.2)								
**Education level, n (%)**								7.344 (5)^a^		—		—		.20
	Illiterate			4 (5.9)		6 (8.8)								
	Primary school			9 (13.2)		14 (20.6)								
	Junior high school			22 (32.4)		10 (14.7)								
	High school			21 (30.9)		22 (32.4)								
	Bachelor degree			12 (17.6)		15 (22.1)								
	Postgraduate and above			0 (0)		1 (1.5)								
**Have children, n (%)**								—		—		—		—
	Yes			68 (100)		68 (100)								
	No			0 (0)		0 (0)								
**Smoke, n (%)**								4.946 (3) ^a^		—		—		.18
	Yes, smoker			11 (16.2)		19 (27.9)								
	No, never smoke			38 (55.9)		39 (57.4)								
	Used smoke, quite now			13 (19.1)		7 (10.3)								
	Occasional smoker			6 (8.8)		3 (4.4)								
Smoke year, median (IQR)				0 (0-30)		0 (0-34.3)		—		—		–0.024^d^		.98
**Drink, n (%)**								2.079 (3)^a^		—		—		.56
	Yes, drinker			8 (11.8)		14 (20.6)								
	No, never drink			35 (51.5)		33 (48.5)								
	Used drink, quite now			9 (13.2)		7 (10.3)								
	Occasional drinker			16 (23.5)		14 (20.6)								
Drink year, median (IQR)				0 (0-39.3)		15 (0-40)		—		—		–0.398^d^		.69
**Personal income, n (%)**								0.256 (1)^a^		—		—		.61
	Have income			58 (85.3)		60 (88.2)								
	No income			10 (14.7)		8 (11.8)								
Personal income (RMB)^f,g^, median (IQR)				4000 (2800-5375)		4000 (2850-5375)		—		—		–0.264^d^		.79
**Household income, n (%)**								0.000 (1)^b^		—		—		>.99
	Have income			66 (97.1)		66 (97.1)								
	No income			2 (2.9)		2 (2.9)								
Household income (RMB)^f,g^, median (IQR)				8000 (7000-10,750)		8000 (6000-10,000)		—		—		–0.389^d^		.70
**Occupational status, n (%)**								1.578 (3)^a^		—		—		.66
	Employed			9 (13.2)		10 (14.7)								
	Unemployed			6 (8.8)		8 (11.8)								
	Retired			40 (58.8)		42 (61.8)								
	Others			13 (19.1)		8 (11.8)								
**Medical payment method, n (%)**								0.342 (3)^a^		—		—		.93
	Self-paid			1 (1.5)		2 (2.9)								
	Medical insurance			61 (89.7)		60 (88.2)								
	Commercial insurance			5 (7.4)		5 (7.4)								
	Others			1 (1.5)		1 (1.5)								
**BMI (kg/m^2^), mean (SD)**				24.04 (3.475)		24.08 (3.428)		—		–0.066 (134)^c^		—		.65
	<18.5			4 (5.9)		4 (5.9)								
	18.5-23.9			30 (44.1)		27 (39.7)								
	24-27.9			27 (39.7)		29 (42.6)								
	≥28			7 (10.3)		8 (11.8)								
**Smartphone usage, n (%)**								1.225 (1)^a^		—		—		.27
	Yes			53 (77.9)		58 (85.3)								
	No			15 (22.1)		10 (14.7)								
T2DM duration, mean (SD)				14.76 (8.381)		14.19 (7.658)		—		–0.063 (134)^c^		—		.55
**Family history, n (%)**								0.353 (2)^a^		—		—		.84
	Yes			23 (33.8)		26 (38.2)								
	No			28 (41.2)		25 (36.8)								
	Do not know			17 (25.0)		17 (25.0)								
**Family member has diabetes mellitus, n (%)**								6.543 (6)^a^		—		—		.37
	No			26 (33.3)		23 (29.5)								
	Father			9 (11.5)		17 (21.8)								
	Mother			10 (12.8)		10 (12.8)								
	Sibling			11 (14.1)		5 (6.4)								
	Grandparents			2 (2.6)		5 (6.4)								
	Do not know			18 (23.1)		17 (21.8)								
	Other relative			2 (2.6)		1 (1.3)								
**Combination disease**														
	**Hypertension, n (%)**							0.034 (1)^a^		—		—		.85
		No	21 (30.9)		22 (32.4)									
		Yes	47 (69.1)		46 (67.6)									
	**Coronary heart disease, n (%)**							0.873 (1)^a^		—		—		.35
		No	50 (73.5)		45 (66.2)									
		Yes	18 (26.5)		23 (33.8)									
	**Chronic heart failure, n (%)**							0.831 (1)^b^		—		—		.36
		No	64 (94.1)		67 (98.5)									
		Yes	4 (5.0)		1 (1.5)									
	**Stroke, n (%)**							0.133 (1)^b^		—		—		.71
		No	63 (92.6)		65 (95.6)									
		Yes	5 (7.4)		3 (4.4)									
	**Kidney disease, n (%)**							0.831 (1)^b^		—		—		.36
		No	64 (94.1)		67 (98.5)									
		Yes	4 (5.9)		1 (1.5)									
	**Other diseases, n (%)**							0.067 (1)^a^		—		—		.80
		No	60 (88.2)		59 (86.8)									
		Yes	8 (11.8)		9 (13.2)									
	**Type of medication, n (%)**							0.216 (2)^a^		—		—		.90
		Oral	37 (54.4)		35 (53.3)									
		Insulin	0 (0)		0 (0)									
		Both	28 (41.2)		29 (42.6)									
		Others	3 (4.4)		4 (5.9)									
	Kind of medication, median (IQR)			3.0 (3-4.8)		3.0 (2-5)		—		—		–0.401^d^		.69
**Adverse reactions**														
	**Hypoglycemia, n (%)**							0.324 (1)^a^		—		—		.57
		No	47 (69.1)		50 (73.5)									
		Yes	21 (30.9)		16 (26.5)									
	**Digestive system, n (%)**							0.471 (1)^a^		—		—		.49
		No	55 (80.9)		58 (85.3)									
		Yes	13 (10.1)		10 (14.7)									
	**Chronic heart failure, n (%)**							0.133 (1)^b^		—		—		.72
		No	63 (92.6)		65 (95.6)									
		Yes	5 (7.4)		3 (4.4)									
	**Allergic reaction, n (%)**							0.000 (1)^b^		—		—		>.99
		No	66 (97.1)		65 (97.1)									
		Yes	2 (2.9)		2 (2.9)									
	**Liver damage, n (%)**							1.681 (1)^a^		—		—		.20
		No	62 (83.8)		69 (92.0)									
		Yes	12 (16.2)		6 (8.0)									
	**Kidney damage, n (%)**							0.319 (1)^a^		—		—		.57
		No	60 (88.2)		62 (91.2)									
		Yes	8 (11.8)		6 (8.8)									
	**Others diseases, n (%)**							0.000 (1)^b^		—		—		>.99
		No	66 (97.1)		65 (95.6)									
		Yes	2 (2.9)		3 (4.4)									

^a^Pearson chi-square.

^b^Continuity correction.

^c^Independent sample *t* test.

^d^Mann-Whitney *U* test.

^e^Not applicable.

^f^1 RMB ≈ RM 0.61 (November 08, 2024).

^g^1 RMB ≈ US $0.14 (January 07, 2026).

#### Medication Adherence, Medication Knowledge, Medication Attitude, Medication Behavior, Medication Belief, and Social Support Between the IG and CG at Baseline

There were no significant differences between the IG and CG of medication adherence (23.71, SD 5.725 vs 24.01, SD 5.700; t_134_=–0.315; *P*=.820), medication knowledge (63.41, SD 19.145 vs 62.37, SD 20.202; t_134_=0.308; *P*=.547), medication attitude (35.76, SD 3.801 vs 36.15, SD 4.129; t_134_=–0.562; *P*=.568), medication behavior (29.62, SD 4.381 vs 29.13, SD 4.029; t_134_=0.672; *P*=.468), medication belief (3.00, SD 3.515 vs 2.94, SD 3.777; t_134_=0.094; *P*=.520) and social support (69.62, SD 11.402 vs 68.22, SD 10.935; t_134_=0.729; *P*=.805) at baseline (Table 3).

**Table 3:** Total score of medication adherence, knowledge, attitude, behavior, belief, and social support between the intervention group (IG) and control group (CG) at baseline among older adults living with type 2 diabetes mellitus in Henan, China.

Outcomes (range)^a^	IG, mean (SD)	CG, mean (SD)	*t* test(*df*)	*P* value^b^
Medication adherence (13 to 39)	23.71 (5.725)	24.01 (5.700)	–0.315 (134)	.82
Medication knowledge (0 to 100)	63.41 (19.145)	62.37 (20.202)	0.308 (134)	.55
Medication attitude (10 to 50)	35.76 (3.801)	36.15 (4.129)	–0.562 (134)	.57
Medication behavior (9 to 40)	29.62 (4.381)	29.13 (4.029)	0.672 (134)	.47
Medication belief (–25 to +25)	3.00 (3.515)	2.94 (3.777)	0.094 (134)	.52
Social support (19 to 95)	69.62 (11.402)	68.22 (10.935)	0.729 (134)	.81

^a^Higher scores of outcomes indicate better medication adherence, knowledge, attitude, behavior, belief, and social support.

^b^*P* value with *P>*.05 indicates that there are no significant differences between the IG and CG for the measured outcomes.

### Outcomes

#### Overview

This study includes 6 outcome variables, including medication adherence, medication knowledge, medication attitude, medication behavior, medication beliefs, and social support. Given the absence of significant effects, no covariates were incorporated into the final model. Generalized estimating equation was used to investigate the changes in outcomes between IG and CG across 3 time points. The results are shown in Table 4.

**Table 4:** Comparison of medication adherence, medication knowledge, medication attitude, medication behavior, medication belief, and social support scores between the intervention group (IG) and control group (CG) across time points using generalized estimating equations among older adults living with type 2 diabetes mellitus in Henan, China.

Variable			Medication knowledge^a^			Medication attitude^b^					Social support^c^					Medication belief^d^				Medication behavior^e^				Medication adherence^f^	
			β (95% CI)	*P* value		β (95% CI)		*P* value		β (95% CI)			*P* value		β (95% CI)		*P* value		β (95% CI)		*P* value		β (95% CI)		*P* value
**Group effect**																									
	CG	Ref^g^		Ref	Ref		Ref		Ref			Ref		Ref			Ref	Ref			Ref	Ref			Ref
	IG	7.887 (3.428 to 12.345)		.001^h^	2.465 (1.466 to 3.464)		<.001^h^		1.277 (–4.771 to 7.352)			.679		1.845 (1.078 to 2.613)			<.001^h^	2.731 (1.639 to 3.823)			<.001^h^	2.288 (0.681 to 3.894)			.005^h^
**Time effect**																									
	T0^i^	Ref		Ref	Ref		Ref		Ref			Ref		Ref			Ref	Ref			Ref	Ref			Ref
	T1^j^	7.093 (4.564 to 9.623)		<.001^h^	3.103 (2.370 to 3.836)		<.001^h^		0.397 (–0.564 to 1.358)			.418		1.632 (1.125 to 2.140)			<.001^h^	2.441 (1.848 to 3.034)			<.001^h^	2.601 (1.858 to 3.363)			<.001^h^
	T2^k^	4.844 (2.462 to 7.227)		<.001^h^	1.662 (1.059 to 2.264)		<.001^h^		–0.574 (–1.491 to 0.344)			.221		0.588 (0.062 to 1.114)			.028^h^	1.125 (0.586 to 1.664)			<.001^h^	1.684 (0.958 to 2.409)			<.001^h^
**Timepoint**×**Group effect**																									
	T0 × CG	Ref		Ref	Ref		Ref		Ref			Ref		Ref			Ref	Ref			Ref	Ref			Ref
	T1 × IG	11.592 (6.923 to 16.260)		<.001^h^	5.467 (4.531 to 6.763)		<.001^h^		0.008 (–1.834 to 2.011)			.928		2.882 (1.990 to 3.775)			<.001^h^	4.176 (3.220 to 5.133)			<.001^h^	4.956 (3.702 to 6.210)			<.001^h^
	T2× IG	6.055 (1.401 to 10.710)		.011^h^	3.059 (1.969 to 4.149)		<.001^h^		–0.147 (–1.982 to 1.688)			.875		1.882 (0.880 to 2.885)			<.001^h^	2.426 (1.429 to 3.424)			<.001^h^	3.691 (2.379 to 5.003)			<.001^h^

^a^Group: QIC=105,178.155, QICC=105,173.944, time: QIC=106,219.108, QICC=106,219.108, time × group: QIC=99,054.337, QICC=99,054.337.

^b^Group: QIC=5783.910, QICC=5780.419, time: QIC=5787.824, QICC=5787.824, time × group: QIC=4587.706, QICC=4687.706.

^c^Group: QIC=37,306.144, QICC=36,267.594, time: QIC=37,378.507, QICC=37,378.597, time × group: QIC=37,198.191, QICC=37,198.191.

^d^Group: QIC=3561.609, QICC=3558.406 time: QIC=3643.971, QICC=3643.971, time × group: QIC=3227.618, QICC=3227.618.

^e^Group: QIC=6009.207, QICC=6004.590, time: QIC=6301.456, QICC=6301.456, time × group: QIC=5304.176, QICC=5304.176.

^f^Group: QIC=11,557.678, QICC=11,551.984 time: QIC=11,731.963, QICC=11,731.963, time × group: QIC=10,617.505, QICC=10,617.505.

^g^Ref: Reference group.

^h^Significant at *P*<.05.

^i^T0: baseline.

^j^T1: postintervention.

^k^T2: 3-month follow up.

#### Primary Outcome

The IG exhibited a significantly higher medication adherence score compared with the CG, with a mean difference of 2.288 points (β=2.288, 95% CI 0.681-3.894, *P*=.005). Participants demonstrated significant improvements in medication adherence both immediately after the intervention (β=2.610, 95% CI 1.858-3.363, *P*<.001) and at the 3-month follow-up (β=1.684, 95% CI 0.958-2.409, *P*<.001) when compared with baseline. The IG showed a significantly higher medication adherence score postintervention, with a difference of 4.956 points compared with the CG at baseline (β=4.956, 95% CI 3.702-6.210, *P*<.001). At the 3-month follow-up, the IG maintained a significantly higher score than the CG at baseline, with a difference of 3.691 points (β=3.691, 95% CI 2.379-5.003, *P*<.001).

#### Secondary Outcomes

Compared with the CG, the IG showed a significant improvement in medication knowledge score, with a postintervention score higher by 7.887 points (β=7.887, 95% CI 3.428-12.345, *P*=.001). Compared with baseline, the medication knowledge score of the participants increased by 7.093 points after the intervention and by 4.844 points at the 3-month follow-up (β=7.093, 95% CI 4.564-9.623, *P*<.001; β=4.844, 95% CI 2.462–7.227, *P*<.001). After the intervention, the medication knowledge score of the IG group was significantly higher than the CG group at baseline (β=11.592, 95% CI 6.923-16.260, *P*<.001). At the 3-month follow-up, the medication knowledge score of the IG group was 6.055 points higher than that of the CG group at baseline (β=6.055, 95% CI 1.401-10.710, *P*=.011).

The medication attitude score of the IG was significantly higher than that of CG, with a difference of 2.465 points (β=2.465, 95% CI 1.466-3.464, *P*<.001). Compared with the baseline, the medication attitude score of the participants significantly increased by 3.103 points after the intervention and by 1.662 points at the 3-month follow-up, respectively (β=3.103, 95% CI 2.370-3.836, *P*<.001; β=1.662, 95% CI 1.059-2.264, *P*<.001). After the intervention, the medication attitude score of the IG group was significantly higher than that of the CG group at baseline, with a difference of 5.467 points (β=5.467, 95% CI 4.531-6.763, *P*<.001). At the 3-month follow-up, the medication attitude score of the IG group was 3.059 points higher than that of the CG group at baseline (β=3.059, 95% CI 1.969-4.149, *P*<.001).

The IG showed a significantly higher medication behavior score compared with the CG, with a mean difference of 2.731 points (β=2.731, 95% CI 1.639-3.823, *P*<.001). Participants demonstrated significant improvements in medication behavior relative to baseline, both immediately after the intervention (β=2.441, 95% CI 1.848-3.034, *P*<.001) and at the 3-month follow-up (β=1.125, 95% CI 0.586-1.664, *P*<.001). Postintervention, the IG scored 4.176 points higher than the CG at baseline (β=4.176, 95% CI 3.220-5.133, *P*<.001). At the 3-month follow-up, the IG also outperformed the CG at baseline, with a difference of 2.426 points (β=2.426, 95% CI 1.429-3.424, *P*<.001).

The IG demonstrated a significantly higher medication belief score than the CG, with an estimated difference of 1.845 points (β=1.845, 95% CI 1.078-2.613, *P*<.001). Compared with baseline, participants showed a significant increase in medication belief score both immediately after the intervention (β=1.632, 95% CI 1.125-2.140, *P*<.001) and at the 3-month follow-up (β=0.588, 95% CI 0.062-1.114, *P*=.028). Furthermore, in the analysis of the interaction between time and group, the IG at postintervention scored significantly higher than the CG at baseline, with a difference of 2.882 points (β=2.882, 95% CI 1.990-3.775, *P*<.001). Similarly, at the 3-month follow-up, the IG maintained a significantly higher score than the CG at baseline (β=1.882, 95% CI 0.880-2.885, *P*<.001).

However, social support did not reach statistical significance in terms of time, group, and the interaction between time and group, in either the IG or CG group (*P*>.05).

## Discussion

### Principal Findings

Research has shown that the IMB model can effectively explain, predict, and improve medication adherence among older adults living with chronic diseases [54,55]. Based on previous research findings, this study combined the IMB model to develop an animated intervention video called “Diabetes Little Helper” which was disseminated through the WeChat platform, further validating the model’s applicability in older adults. As with the results of this study, a quasi-experimental study by Mirzaei-Alavijeh et al [56], based on the IMB model, significantly improved medication adherence in older adults living with T2DM through 6 face-to-face educational sessions, further supporting the effectiveness of the IMB model. This study, through the WeChat platform, disseminates educational information in video format, providing new evidence and intervention pathways for improving medication adherence among older adults living with T2DM.

A notable proportion of participants reported not using smartphones independently. This finding is significant, as it could influence the overall effectiveness of the intervention. However, it is important to note that all participants in this study either owned a smartphone and used WeChat or received assistance from family members to participate. Nonetheless, the involvement of family members could be seen as a limitation, as it may affect the autonomy of older adults and potentially influence their overall engagement with the intervention. Additionally, reliance on family assistance raises concerns about the generalizability and long-term feasibility of the intervention, particularly for older adults who may not have family members available to offer support. To address this, future research could explore alternative strategies to enhance technological support for older adults, such as simplified user interfaces or targeted training programs. These approaches could ensure broader accessibility and improve the effectiveness of similar interventions in diverse populations.

Participants’ medication adherence score showed significant improvement following the intervention, a result that mirrors outcomes seen in other studies using different methods. For instance, mobile apps were used to provide educational information to family members, who in turn helped older adults living with DM complete self-management tasks, pharmacy students conducted motivational interviewing through phone calls to enhance medication adherence, while pharmacists used apps, WeChat public accounts, WeChat groups, and both web-based and offline lectures to improve medication adherence among older adults living with DM [30,31,33]. These interventions effectively increased medication adherence in older adults living with DM, though they also presented certain challenges. Relying on family members to convey information poses the risk of information distortion and places psychological pressure on the family. Dependence on pharmacy students for motivational interviewing might lead to insufficient mastery of communication and problem-solving skills, potentially affecting the interview’s effectiveness. Although pharmacists use a combination of web-based and offline platforms to provide comprehensive services, the use of multiple channels may confuse older adults, especially those less familiar with digital technologies. In contrast, this study directly engaged patients via WeChat, eliminating hierarchical errors in information transmission and avoiding family involvement in the medical process. The professionally designed animated videos ensured the accuracy and coherence of the information, minimizing human bias. Moreover, the approach did not require the development of complex programs or offline services, thus saving both manpower and financial resources.

This study demonstrated that the Diabetes Little Helper video significantly improved participants’ medication knowledge score, which is consistent with previous research findings [57]. Compared with the one-on-one face-to-face educational model used by Yang et al [57], this study overcame the limitations of time and location while also saving human resources. Xiong and Wan [58] also improved older adult participants’ medication knowledge score by sending text and images via the WeChat platform. In comparison, the animated video used in this study not only incorporated the advantages of text and images but also effectively captured patients’ attention through its vivid and engaging format, helping viewers better understand concepts and stimulate cognitive responses. The use of animated videos has been shown to be a more attractive and dynamic method, aiding viewers in gaining a deeper understanding of health education content [59]. Future intervention research could explore how to combine face-to-face education with digital video interventions, leveraging the strengths of both traditional and modern educational methods to further enhance patients’ medication knowledge scores.

Participants’ medication attitudes were effectively improved, as agreed with Chen et al [60], who used a multidimensional, multiscenario intervention approach, which also successfully enhanced medication attitudes. However, their method requires substantial human resources and high costs, making it challenging to implement in regions with limited health care resources. Zhou [61] conducted an intervention through community health service centers, regularly monitoring patients’ medication levels, metabolism, and relevant physiological indicators to guide medication adjustments, which improved medication attitudes in older adults. In contrast, the intervention model in this study overcomes regional and resource limitations through digital means, making it simple and feasible. Each intervention method has its unique advantages and suitable contexts. Future research should flexibly select intervention strategies based on the specific characteristics of hospitals and patients, and integrate multiple approaches to more effectively improve medication attitudes in older adults.

The intervention also had a positive impact on improving participants’ medication behavior score, in line with the findings of Wang et al [62], whose study increased medication adherence through personalized plans and continuous optimization. However, their approach relied heavily on frequent face-to-face reminders, which may have provoked resistance from some patients. In this study, patients were able to choose their learning time and frequency, which reduced resistance and improved psychological acceptance. Moreover, the intervention program by Wan et al [63] required 50 participants to provide more tailored health education and medication management. Although it also led to some improvement in medication behavior, the management process was relatively complex and added additional costs. Moreover, staff turnover, absenteeism, and communication difficulties could negatively impact the effectiveness of the intervention.

The results of this study also indicate a significant improvement in participants’ medication beliefs, aligning with findings from previous research [64,65]. These studies used interviews or multidisciplinary collaboration (involving family doctors, pharmacists, and nurses) to integrate and guide medication management. However, these approaches are typically limited to stable patients who can attend interviews and are not applicable to older adults living at home. Additionally, team collaboration demands more human resources. However, this study focused on broadening medication knowledge and promoting self-management, encouraging older adults to independently adjust their medication attitudes.

This study did not show a significant effect on improving participants’ social support scores. This finding is consistent with the results of Yang et al [57], who also did not observe significant improvements in social support after using traditional intervention methods, such as face-to-face education, interviews, phone communication, and record-keeping. One possible reason for this is that the development of social support requires sustained intervention over more than 6 months [66]. Additionally, some studies have shown that including education for patients’ family members and facilitating web-based interactive communication effectively improved social support among older adults [67,68]. Future research could integrate interactive modules, such as involving family members in the intervention plan, promoting web-based communication among patients, and combining community nursing, to further strengthen the patient’s social support network and enhance the overall intervention effectiveness.

This study is the first to develop an animated video intervention to improve medication adherence in older adults living with T2DM, expanding the application of this model in medication management for older adults living with DM and providing new insights for chronic disease management. Moreover, this study innovatively used the WeChat public platform to deliver animated video intervention content to older adults living with DM. This approach not only overcomes the time and space limitations of traditional health education but also leverages the widespread use and interactivity of the WeChat platform, enabling continuous health interventions. As a result, it significantly increased the accessibility of intervention content and enhanced patient acceptance. Patients could repeatedly watch the intervention videos in their daily lives, which helped reinforce memory and promote behavior change.

### Limitations and Future Research

While the findings of this study provide valuable insights into the effectiveness of the intervention, several limitations must be acknowledged. First, the reliance on self-reported adherence introduces potential biases, such as over-reporting, underreporting, and recall bias. These biases can compromise the accuracy of adherence measurements and may not fully reflect the participants’ true behaviors. Future studies could incorporate objective measures of adherence, such as electronic monitoring or biomarkers, to reduce these biases and provide a more accurate picture of adherence. Second, while the 3-month follow-up period allowed us to assess the short-term effects of the intervention, it was insufficient to evaluate the long-term sustainability of the intervention’s impact. Future studies should aim for extended follow-up periods to gain a deeper understanding of the long-term benefits and challenges of such interventions. Furthermore, the inability to blind participants due to the visible nature of the intervention video may have introduced bias, as participants were aware of their intervention condition, which could have influenced their responses or behaviors. To mitigate this in future studies, an RCT design with blinding could be implemented, or a different form of intervention that is less visible could be explored.

Adherence monitoring measures, though designed to ensure compliance, may have inadvertently influenced engagement and adherence, thereby introducing additional intervention effects. Future studies should consider monitoring approaches that minimize such influences. In terms of data handling, this study used the LOCF method to address missing data. Although LOCF is computationally simple and commonly used in short-term studies, it assumes that missing data are equivalent to the last observed value, which may lead to an underestimation of variability in the results. If participants drop out due to deteriorating health, LOCF may fail to capture this change, potentially overestimating the intervention effect. Despite these limitations, LOCF was considered an appropriate choice for this study. Future research could consider exploring other advanced methods for handling missing data, such as multiple imputation, to improve the accuracy of data analysis.

### Conclusions

This study evaluated the impact of a WeChat-based Diabetes Little Helper video intervention on medication adherence and medication-related outcomes in older adults living with T2DM. The results indicated that the intervention had a significant positive effect on improving patients’ medication adherence, knowledge, attitude, behavior, and belief. However, it did not significantly enhance the patients’ level of social support. Building on these findings, future research could expand to include more regions and health care institutions and conduct multicenter RCTs to verify the applicability and effectiveness of the Diabetes Little Helper video intervention across different geographic and cultural contexts. This would help to further improve the external validity and reliability of the study’s findings. Moreover, future studies could explore the integration of more advanced health tools, such as smart wristbands and smart pillboxes, to monitor the health status and medication adherence of older adults living with T2DM in real time, thereby providing additional support for personalized interventions and data analysis.

## Appendix

Multimedia Appendix 1 CONSORT eHEALTH checklist (V 1.6.1).

## Abbreviations

CG: control group
CONSORT: Consolidated Standards of Reporting Trials
DM: diabetes mellitus
I-CVI: Item-level Content Validity Index
IG: intervention group
IMB: information–motivation–behavioral skills
LOCF: last observation carried forward
RCT: randomized controlled trial
S-CVI: Scale-level Content Validity Index
T2DM: type 2 diabetes mellitus

## Data Availability

The datasets generated or analyzed during this study are available in the Mendeley data repository.
